# Expression of Circulating MicroRNA-141 in Epithelial Ovarian Cancer

**DOI:** 10.21315/mjms2020.27.6.4

**Published:** 2020-12-01

**Authors:** Akbar Satria Fitriawan, Aprilia Indra Kartika, Siti Nur Chasanah, Teguh Aryandono, Sofia Mubarika Haryana

**Affiliations:** 1 Department of Nursing, Faculty of Health Science, Respati University of Yogyakarta, Yogyakarta, Indonesia; 2 Department of Medical Laboratory Technology, Faculty of Nursing and Health Science, Universitas Muhammadiyah Semarang, Semarang, Indonesia; 3 Department of Biochemistry and Molecular Biology, Faculty of Medicine, Universitas Wahid Hasyim, Semarang, Indonesia; 4 Division of Surgical Oncology, Department of Surgery, Faculty of Medicine, Public Health and Nursing, Universitas Gadjah Mada, Yogyakarta, Indonesia; 5 Department of Histology and Cell Biology, Faculty of Medicine, Public Health and Nursing, Universitas Gadjah Mada, Yogyakarta, Indonesia

**Keywords:** epithelial ovarian cancer, biomarker, plasma, microRNA, miR-141

## Abstract

**Background:**

Epithelial ovarian cancer (EOC) is a lethal disease due to late diagnosis and lack of effective screening methods. MicroRNA (miR/miRNA) plays an important role in ovarian carcinogenesis and may serve as a non-invasive biomarker for EOC. This study aimed to assess miR-141 expression in the blood plasma of patients with EOC and healthy subjects and determine its association with the clinical stage of EOC.

**Methods:**

This cross-sectional study used blood plasma from 30 newly diagnosed untreated patients with EOC and 25 healthy subjects. The mean age was 47.73 (SD = 10.29) years for EOC and 44.48 (SD = 16.14) years for healthy subject. The total RNA was isolated from blood plasma and reversed transcribed to obtain cDNA. The expression of miR-141 was measured by real-time quantitative polymerase chain reaction (qRT-PCR), and calculated using 2^−ΔΔCt^ methods. The data were analysed using Mann-Whitney test.

**Results:**

The expression of miR-141 was upregulated 8.41 fold in the blood plasma of EOC patients compared to healthy controls (*P* < 0.001). Expression of miR-141 in the advanced stage was upregulated 4.2 fold compared to the early stage (*P* < 0.001).

**Conclusion:**

The miR-141 was upregulated in the blood plasma of EOC and associated with an advanced stage of disease, suggesting it has potential as a biomarker for EOC detection.

## Introduction

Ovarian cancer is one of the most common and the most deadly gynecological malignancies among women around the world (1–3), with only 46% survival rate within 5 years after the diagnosis (4, 5). The global incidence and mortality rates of ovarian cancer were 295,414 cases and 184,799 deaths in 2018 (6). According to the histological types, the most frequent ovarian cancer suffered by women is epithelial ovarian cancer (EOC) that accounts for around 80%–90% of all ovarian malignancy (7, 8).

EOC develops from the malignant transformation of the epithelial cells of the ovarian surface, peritoneum, or fallopian tube (9). EOC is not a single entity but a heterogeneous disease and can be divided into several histological subtypes based on the histopathology, immunohistochemistry, genetic alteration and the molecular mechanisms underlying the carcinogenesis process (10). Recently, five types of EOC have been identified: high-grade serous carcinomas (HGSC), low-grade serous carcinoma (LGSC), endometrioid carcinomas, clear-cell carcinomas and mucinous carcinomas (10, 11). These subtypes of EOC have different risk factors, pathogenesis and responses to treatment (11).

The International Federation of Gynecology and Obstetrics (FIGO) staging system is widely used in clinical practice to determine the clinical stage of ovarian cancer (12). The FIGO staging system classified ovarian cancer into four clinical stages. At stage I, cancer is confined to ovaries or fallopian tube, whereas at stage II, cancer involves one or both ovaries and has spread to other pelvic organs (10). Stages I and II ovarian cancer are considered as the early stage and associated with a high survival rate (12). Stage III is characterised by cancer spreading to the peritoneum outside the pelvis and/or metastasis to the retroperitoneal lymph nodes, and stage IV is defined as a distant metastatic disease (10). Stages III and IV are considered as the advanced disease and are associated with poor prognosis (13).

One of the main factors contributing to the high mortality rate is the advanced stage of the disease at the time of diagnosis (14). More than 80% of EOC cases are diagnosed at an advanced stage due to the asymptomatic nature of EOC at early stages and lack of sensitive screening methods (1, 14, 15). This condition leads to the difficulties to determine whether there are malignant or benign ovarian masses. Therefore, the diagnosis is determined after the invasive histological examination (16). Patients with advanced-stages of EOC have a 5-year relative survival rate of less than 30%, while patients with early-stage disease have a 5-year survival rate of more than 90% (17–19). The difficulty in discovering ovarian cancer at an early stage is a major factor contributing to poor clinical outcomes.

Current strategies and methods for EOC detection include the pelvic examination, transvaginal ultrasonography (TVS) and measurement of several molecular biomarkers (16, 20, 21). The pelvic examination has limited accuracy for detecting ovarian cancer and this finding could represent conditions other than ovarian cancer (22). Many small but potentially life-threatening tumours cannot be palpated and pelvic examination cannot differentiate benign or malignant conditions. Based on a previous systematic review, pelvic examination has a poor accuracy to screen for ovarian cancer and to distinguish benign or malignant masses (23). Generally, detection by pelvic examination reveals advanced disease, suggesting the limitation of this method to detect the early-stage disease. The TVS method is accurate in detecting abnormalities in ovarian volume and morphology but is less reliable in distinguishing the benign or malignant condition. When TVS is used as a single screening method, it has a good sensitivity but has a low specificity. Accordingly, molecular biomarkers must be used together with TVS to detect ovarian cancer (24).

Recently, only CA-125 and human epididymis protein 4 (HE4) have been approved by the Food and Drug Administration (FDA) for monitoring treatment and detecting disease recurrence (25). The serum CA-125, HE4 and other serum biomarkers such as apolipoprotein A1, transthyretin, transferrin and β2-macroglobulin, are not adequately sensitive nor specific as early detection biomarkers (16, 26, 27). The identification and validation of the early detection biomarkers for ovarian cancer with high sensitivity and specificity are urgently needed, which would permit the development of minimally invasive screening methods for detecting an early onset of the disease (20).

MicroRNA (miR/miRNA) are small non-coding RNA molecules with a length of 19–24 nucleotides, phylogenetically conserved in various species, that have a role in the regulation of protein-coding gene expression at post-transcription levels (28–30). MiRNA regulate the gene expression through binding to target messenger RNA (mRNA) and cause either the mRNA degradation or the inhibition of translation (30, 31). MiRNA binds to the target mRNA then guides the RNA induced silencing complex (RISC) to the target mRNA so that it will cause translation inhibition and/or degradation of the target mRNA (32). Single miRNA binds to 100 different target mRNAs and it has been believed that miRNAs regulate up to 30% of the protein-coding genes in the human genome (33). The capacity of miRNA to regulate the majority of genes in humans is essential in regulating various key cellular phenotypes, such as proliferation, apoptosis, metastasis, angiogenesis and embryonal development (29, 30, 32). The alteration of miRNA contributes to the development of different diseases, including the development and progression of cancer (34).

MiRNA are also found secreted out of the cells as cell-free miRNA, packaged in exosomes, micro-vesicles or RNA-binding-proteins, that provide protection from RNases and serve as a new mode in cell communication (35). Cell-free miRNA also enable the communication between cells within the tumour microenvironment, thereby influencing tumourigenesis. Previous studies have demonstrated that miRNAs are stable in serum and plasma, so their expression can be quantified in blood plasma samples (35, 36). Additionally, it has been demonstrated that the miRNA expression profile in plasma responds to the alterations under different physiological and pathological conditions. Since the miRNA are also secreted from cancer cells, the deregulation is associated with tumour development and progression, and they represent not only therapeutic targets but also promising minimal invasive biomarkers for diagnosis and prognosis of cancer (35).

The miR-141 is a member of the miR-200 family, which is a family of miRNA that maintains epithelial cell integrity by inhibiting epithelial to mesenchymal transition (EMT). Several studies have reported the upregulation of miR-141 in cancers, such as colorectal cancer (34), prostate cancer (37, 38), bladder cancer (39, 40) and ovarian cancer (18, 27, 41, 42) suggesting miR-141 may act as an oncogene miRNA. Interestingly, other studies have reported the down regulation of miR-141 in various human cancers such as colorectal cancer (43), hepatocellular carcinoma (44) and ovarian cancer (45), suggesting that this miR also acts as a tumour suppressor miRNA.

Since there are different results in the miR-141 expression among studies, we aimed to evaluate the expression of miR-141 in the blood plasma of patients with EOC and healthy subjects. The results could be helpful to evaluate miR-141 as a potential novel screening biomarker for EOC and other ovarian malignancies.

## Materials and Methods

### Clinical Sample Collection

This observational, analytical study used a cross-sectional design. All eligible patients with EOC diagnosed clinically, and confirmed pathologically, irrespective of age were included in this study. If patients had one of the following criteria, they were excluded from the study: i) patients with other organ cancers; ii) history of chemotherapy or radiotherapy; iii) pregnancy; iv) diabetes mellitus; and/or v) impaired renal function/diagnosed with renal disease.

The sample size calculation was obtained from the following formula (46):

$$\text{Sample size}=\frac{r+1}{r}\frac{{\text{SD}}^{2}{\left(\text{ZB}+\text{Z}\alpha /2\right)}^{2}}{d^{2}}$$

*r* = Sample size ratio between EOC group and healthy control group is 1
SD = Standard deviation of miR-141 expression in the blood plasma of cancer patient. Based on the previous study (40), standard deviation value was 2.56
*d* = Expected mean difference of miR-141 expression between cancer and healthy control. Based on the previous study (40), the mean difference was 2.17
Zα/2 = Standard normal variate at 5% Type I error is 1.96
ZB = Standard normal variate for 80% power is 0.84

Based on formula above, the minimum sample size for each group was 25. In this study, blood plasma samples were collected from 30 newly diagnosed untreated EOC patients at Dr Sardjito General Hospital (Yogyakarta, Indonesia) from September 2015 to May 2016. Blood plasma samples obtained from 25 healthy women were used as the control group.

Informed consent was obtained from each patient before the blood was collected. Five millilitres of blood samples were withdrawn from a peripheral venous puncture. The blood was collected using an ethylenediaminetetraacetic acid (EDTA) tube. Immediately after sample collection, the blood samples were centrifuged at 1,500 rpm for 10 min at 4 °C. Plasma was transferred into 1.5 mL RNase-free tubes using a tip filter and then the plasma was stored at −80 °C until further use.

### Total RNA Extraction from Blood Plasma

Total RNA extraction from 200 μL of blood plasma was performed according to the manufacturer’s instructions of miRCURY RNA Isolation Kit-Biofluid (Cat No.300112, Exiqon, Denmark). Then, the concentration and purity were determined using a nanodrop spectrophotometer. The RNA was stored in a refrigerator −80 °C for reuse.

### Reverse Transcription and Real-Time Quantitative Polymerase Chain Reaction (qRT-PCR)

A total of 4 μL of RNA isolated from plasma was reverse transcribed into complementary DNA (cDNA) using the universal cDNA synthesis kit II (Cat No.203301, Exiqon). The reverse transcription reaction was done by mixing the RNA, 5× reaction buffer, nuclease-free water, enzyme mix and spike in (sp6) for a total volume of 20 μL. Then, it was incubated at 42 °C for 60 min, with reverse transcriptase inactivation at 95 °C for 5 min, and cooling down at 4 °C. The cDNA was stored in a refrigerator at −20 °C.

The real-time quantitative polymerase chain reaction (qRT-PCR) assay was accomplished using ExiLent SYBR Green master mix, 2.5 mL (Cat No. 203 402, Exiqon) and LNA miRNA PCR Primer Set (Exiqon) on Bio-Rad CFX96 Real-time Thermal Cycler System (Bio-Rad, USA).

The primer sequence for miR-141 was (F: 5′-TGGGTCCATCTTCCAGTA-3′, R: 5′-GGGAGCCATCTTTACCAG-3′) (47). The primer sequence for miR-16 was (F: 5′-TAGCAGCACGTAAATATTGGCG-3′, R: 5′-TGCGTGTCGTGGAGTC-3′) (48).

The cDNA was diluted with RNase-free water at a ratio of 1:80 (1 μL cDNA with 80 μL RNase-free water). After that, 4 μL cDNA was then added with 5 μL SYBR Green master mix and 1 μL miRNA specific primer for a total volume 10 μL. The qRT-PCR was performed with the following conditions: early denaturation at 95 °C for 10 min, followed by 40 cycles 95 °C for 10 s, 60 °C for 1 min ramp-rate 1.6 °C/s optical read and analysed melting curve. The cycle of threshold (Ct) values were normalised using miR-16 as the reference gene. To assess the contamination, the no template control (NTC) was included at each qRT-PCR reaction.

The relative miR-141 expression was calculated using 2^−ΔΔCt^ (49–51). The differences of copy number of miR-141 between the EOC group and healthy control group was calculated using the formula ΔΔCt miR-141 = (Ct miR-141−Ct miR-16) of EOC group-(Ct miR-141–Ct miR-16) of the healthy control group. The differences of copy number of miR-141 between advanced stage and early stage EOC were calculated using the formula ΔΔCt miR-141 = (Ct miR-141−Ct miR-16) advanced stage group-(Ct miR-141−Ct miR-16) early-stage group.

### Statistical Analysis

Statistical analyses were performed using SPSS 23 (IBM Corp., Chicago). All numeric data were tested using Kolmogorov-Smirnov to assess the data distribution. We reported the data as mean and SD for normally distributed data, while the non-normally distributed data were reported as median and interquartile range (IQR). The age difference between patients with EOC and healthy controls was calculated using independent sample *t*-test because age distribution is normal based on Kolmogorov-Smirnov test (*P >* 0.05). The differences in expression level of miR-141 between patients with EOC and healthy controls were calculated using Mann-Whitney test, because the distribution of ΔCT miR-141 value was abnormal (Kolmogorov-Smirnov test, *P* < 0.05). The differences of expression level of miR-141 between early stage and advanced stage EOC were calculated using Mann-Whitney test, because the distribution of ΔCT miR-141 value was abnormal based on Kolmogorov-Smirnov test calculation (*P* < 0.05). The differences in expression level of miR-141 between Type I EOC and Type II EOC were calculated using Mann-Whitney test, because the distribution of ΔCT miR-141 value was abnormal (Kolmogorov-Smirnov test, *P* < 0.05). The *P*-value of less than 0.05 was considered statistically significant.

## Results

### Patient Characteristics

From September 2015 to May 2016, 30 female patients with histologically proven EOC were consecutively recruited for this study. A total of 25 healthy females was also recruited as a control group. Patients’ characteristics are presented in Table 1.

The mean age was 47.73 years (SD = 10.29 years) in the EOC group and 44.48 years (SD = 16.14 years) in the healthy control group. There was no significant difference in age between the EOC and control group (*t*-test, *P* = 0.533). In this study, the clinical stage of EOC was determined based on the FIGO staging system. Based on the clinical stage, 15 patients (50%) were diagnosed at an advanced stage, with 12 patients (40%) diagnosed with FIGO stage III and 3 patients (10%) diagnosed with FIGO stage IV. Based on histological types, serous and mucinous EOC were the most common types diagnosed in this study. Results showed 43.3% of patients were diagnosed with serous EOC, 36.7% of patients diagnosed with mucinous EOC, 6.7% of patients diagnosed with endometrioid EOC and 13.3% of patients diagnosed with clear cell EOC. All of the EOC patients in this study have a CA-125 level higher than the normal value (> 35 U/mL).

### Relative Expression of miR-141 in the Blood Plasma of EOC and Healthy Subjects

The median ΔCt miR-141 was 9.37 (IQR = 2.53) in the EOC group and 11.64 (IQR = 1.49) in the healthy control group (Table 2). The higher ΔCt indicated the lower miR-141 expression; therefore, the relative expression of plasma miR-141 was upregulated significantly in the EOC group compared to the control group (*P* < 0.001). The relative expression of miR-141 in the blood plasma of EOC patients was upregulated 8.41 fold compared to the control group.

### Relative Expression of miR-141 in the Blood Plasma Based on Clinical Stage

To determine the essential role of plasma miR-141 in the progression of EOC, we examined plasma miR-141 expression based on the clinical stage. For this purpose, we classified the clinical stage into two groups: early-stage (FIGO stages I and II) and advanced-stage (FIGO stages III and IV). As indicated in Table 3, the median ΔCt miR-141 was 10.57 (IQR = 1.86) in the early stage, and 8.5 (IQR = 1.78) in the advanced stage (*P* < 0.001). This result demonstrated that the expression of miR-141 was elevated significantly in the advanced stage compared to the early stage. The relative expression of plasma miR-141 in advanced stage EOC patients was upregulated 4.2 fold compared to early stage EOC patients.

### Relative Expression of miR-141 in the Blood Plasma between Types I and II EOC

Recent morphologic, immuno-histochemistry and molecular genetics studies suggest that there are two types of EOC: Type I and Type II (52). The histological Type I tumours consist of low-grade serous, mucinous, endometrioid, clear cell and transitional cell carcinomas subtypes. Meanwhile, Type II tumours comprise high-grade serous carcinomas, undifferentiated carcinomas and carcinosarcomas subtypes (52). In this study, we compared the expression of plasma miR-141 between Type I and Type II EOC. The differences in the median, IQR, and *P*-values of miR-141 between Type I and Type II EOC are presented in Table 4. The median ΔCt miR-141 was 9.75 (IQR = 2.88) in the Type I group and 9.19 (IQR = 2.61) in the Type II group (*P* = 0.592). This result suggests that the relative expression of plasma miR-141 is not significantly different between Type I and Type II EOC.

## Discussion

Ovarian cancer is ranked as the eighth major contributor to death in women due to cancer (4), which is a result of the generally ineffective detection and unfavorable prognosis of ovarian cancer (1, 14, 15). Around 80%–90% of all ovarian cancers are caused by EOC (7, 8). In this study, we ascertained 30 newly diagnosed EOC patients between September 2015 and May 2016. The advanced stage of the time of diagnosis leads to a high mortality rate (14). Our study demonstrated that half of the EOC patients were diagnosed at an advanced stage. Early-diagnosis of EOC is difficult to achieve due to the asymptomatic nature of EOC at the early stage and lack of sensitive screening methods. Albeit, CA-125 is considered as the most promising biomarkers in serum, the sensitivity and specificity are not sufficient to detect cancer at an early stage (16, 26, 27, 51). Less than half of the total cases of ovarian cancer are diagnosed effectively because of its limited specificity and sensitivity (27).

Based on the histological subtype of EOC, serous is the most common type found, followed by mucinous. The serous subtype of EOC is the most common histological type in the world, especially in Western countries (53). Data obtained from Cipto Mangunkusumo Hospital, Jakarta, Indonesia, found that there were 397 cases of EOC from 2010 to 2017, with serous type cancer as the most common type. Purbadi et al. (54) demonstrated that serous is the second most common EOC type after mucinous.

Minimal invasive biomarkers with high sensitivity and specificity for women with EOC are urgently required. In this study, we found that the relative expression of plasma miR-141 was upregulated significantly in patients with EOC compared to healthy subjects. Our results of the circulating miR-141 expression levels are consistent with a previous study, which confirmed that the miR-141 was highly-overexpressed in patients with EOC (27). Other previous studies using tissue samples also confirmed that miR-141 was overexpressed in patients with EOC (55, 56) suggesting that the deregulation of miR-141 expression in plasma is in line with the deregulation of miR-141 in EOC tissues. We found that the relative expression of plasma miR-141 was upregulated significantly in the advanced stage compared to the early stage. This study’s results are consistent with a previous research, which confirmed that the miR-141 was highly-overexpressed in advanced stage disease (27).

Our results suggested that miR-141 acts as onco-miR in EOC. Some studies have tried to elucidate the underlying mechanism of the miR-141 overexpression induced cancer phenotype. An epigenetic mechanism such as DNA methylation may cause deregulation of miRNA expression in cancer (57). The DNA hypomethylation is an epigenetic alteration that plays an important role in cancer. The cytosine-phosphodiester-guanine (CpG) island of the various promoters of the gene is normally methylated in normal tissue and the loss of DNA methylation in these CpG islands causes activation of the gene, which is a phenomena found in several cancers (58). Early studies showed that DNA hypomethylation occurs in the promoters of oncogenes, which coincides with the loss of 5-methylcytosine and which is mostly found in metastatic tumours (59). Previous studies demonstrated that the DNA hypomethylation at the promoters of the onco-miR genes stimulate onco-miR transcriptional activation and leads to cancer progression (60). The promoters of genes encoding miR-106b, miR-25, miR-93, miR-23a and miR-27a are hypomethylated and associated with upregulation of these miRs in human hepatocellular carcinoma (61). Promoter hypomethylation of the miR-21, miR-34a and miR-155 causes upregulation of these miRs in chronic lymphocytic lymphoma, and leads to cancer progression (62). A study by Lynch et al. (63) in 2016 found that knockdown of DNA methyltransferase 1 (DNMT1) induces the upregulation of miR-141 expression in cancer, suggesting DNMT1 plays an important role in epigenetic regulation of miR-141. One study found that the expressions of DNMT1, DNMT3A, DNMT3B and DNMT3L are significantly downregulated in EOC compared to the normal ovary (59). The overexpression of miR-141 found in this study could be caused by DNA hypomethylation-mediated DNMT1 downregulation.

Bromodomain containing 7 (BRD7) is a transcription factor that acts as a tumour suppressor protein in various cancers, including in ovarian cancer (64, 65). Overexpression of BRD7 was associated with the down-regulation of primary, precursor and mature miRNA-141 suggesting that BRD7 can negatively regulate the promoter activity of miR-141 (65). The previous research conducted by Park et al. (64) in 2016 suggested that the expression of BRD7 was downregulated significantly in EOC which may underlie the upregulation of miR-141 expression in EOC.

Single miRNA can regulate multiple protein-coding genes, making miRNA exceptionally important regulators in a variety of cellular phenotypes (32). A recent study using luciferase reporter assay demonstrated that miR-141 directly targeting mRNA phosphatase and tensin homolog (PTEN) and overexpression of miR-141 caused a decrease of PTEN expression (66). PTEN is known as an intracellular phosphatase enzyme that inhibits the phosphatidylinositol 3-kinase (PI3K)/protein kinase B (AKT) signaling pathway by catalysing the dephosphorylation of phosphatidylinositol 3,4,5-triphosphate (PIP3) into phosphatidylinositol 4,5-bisphosphate (PIP2) (67). This mechanism suggests that miR-141 induced PTEN downregulation may contribute to increased AKT activation and development of cancer phenotypes. Ishibashi et al. (68) demonstrated that miR-141 directly targets pleckstrin homology domain leucine-rich repeat protein phosphatase-2 (PHLPP2). PHLPP2 is known as a protein phosphatase enzyme that negatively regulated the PI3K-AKT signaling pathway by catalysing dephosphorylation of ser-473 on AKT resulting in the inactivation of AKT kinase activity (69). Another study found that overexpression inhibits apoptosis by targeting the Kruppel-like factor 12 (KLF12) gene in ovarian cancer cells. Overexpression of miR-141 in ovarian cancer cells also resulted in cisplatin resistance by directly targeting Kelch-like ECH-associated protein 1 (KEAP1) (70).

In this study, we found that miR-141 expression was elevated in advanced stage EOC. A study by Mak et al. (71) found that the upregulation of miR-141 increased anoikis resistance in vitro through the KLF12/Sp1/Survivin axis. Anoikis is apoptosis that occurs when cells detach from the extracellular matrix (72). Anoikis resistance is an important mechanism in cancer metastasis because cancer cells that acquire anoikis resistance can survive after detachment from their primary site and while spreading to the distant organ through blood circulation (72).

Effective screening strategy for EOC must not only have a high sensitivity for early-stage disease (> 75%) but must also have a very high specificity (99.6%) to achieve an acceptable positive predictive value (PPV). Currently, CA-125 is widely used as a biomarker in clinical settings both for detection and disease monitoring in EOC. Although CA-125 is upregulated in the serum of most women with ovarian cancer, around 50% of stage I ovarian cancer cases have serum CA-125 level below the conventional cut-off of 35 U/mL (73). Besides that, there have also been reports of elevated serum CA-125 level in some cancers (74), liver disease (75), congestive heart failure (76) and also in infectious diseases such as tuberculosis (77, 78). Our study demonstrates that miR-141 was upregulated significantly in the blood plasma of patients with EOC. Other studies showed that among 48 miRNAs in ovarian cancer, the miR-141 is upregulated significantly (9.41 fold) compared to healthy controls (79). This finding suggests that circulating miR-141 is a promising biomarker for EOC. However, this study has not assessed the sensitivity and specificity of miR-141 as a detection method. Further studies are needed to examine the sensitivity and specificity of miR-141 when used alone or in combination with other available detection methods.

## Conclusion

In conclusion, this study demonstrated that circulating miR-141 levels were upregulated in patients with EOC compared with the expression levels in healthy subjects. Furthermore, miR-141 expression levels were also upregulated in advanced stage compared to early-stage disease. Our results indicated that circulating miR-141 is a potential minimally invasive biomarker for EOC detection and prognostication.

## Figures and Tables

**Table 1:** Summary of patient characteristics and clinicopathological variables

Characteristic	Patients (*n* = 30) *F* (%)	Controls (*n* = 25) *F* (%)	*P*
Age (years) ∴	47.73 (10.29)	44.48 (16.14)	0.533*
< 45	12 (40%)	13 (52%)	0.373**
≥ 45	18 (60%)	12 (48%)	
FIGO Stage		N/A	
I	13 (43.3%)		
II	2 (6.7%)		
III	12 (40%)		
IV	3 (10%)		
Histology		N/A	
Serous	13 (43.3%)		
Mucinous	11 (36.7%)		
Endometrioid	2 (6.7%)		
Clear cell	4 (13.3%)		
CA-125 (U/mL)		N/A	
0–35	0 (0%)		
> 35	30 (100%)		

Notes:

∴ mean (SD);

* Student *t*-test;

** Chi-square test;

N/A = not available

**Table 2:** Relative expression of miR-141 in blood plasma of EOC and healthy controls

Group	ΔCt miR-141*		ΔΔCt miR-141**	Fold change (2^−ΔΔCt^) in EOC versus controls	*P* (Mann-Whitney test)
	Median	IQR			
EOC	9.37	2.53	−3.08	8.41	< 0.001
Healthy controls	11.64	1.49			

Notes:

* ΔCt miR-141 = (Ct miR-141−Ct miR-16);

** ΔΔCt miR-141 = ΔCt miR-141 EOC-ΔCt miR-141 healthy controls

**Table 3:** Relative expression of miR-141 in early stage and advanced stage EOC

Group	ΔCt miR-141*		ΔΔCt miR-141**	Fold change (2^−ΔΔCt^) in advanced versus early stage	*P* (Mann Whitney test)
	Median	IQR			
Early stage	10.57	1.86	−2.07	4.2	0.001
Advanced stage	8.5	1.78			

Notes:

* ΔCt miR-141 = (Ct miR-141−Ct miR-16);

** ΔΔCt miR-141 = ΔCt miR-141 advanced stage-ΔCt miR-141 early stage

**Table 4:** Relative expression of miR-141 in Type I and Type II EOC

Group	ΔCt miR-141*		ΔΔCt miR-141**	Fold Change (2^−ΔΔCt^) in Type I versus Type II	*P* (Mann-Whitney test)
	Median	IQR			
Type I EOC	9.75	2.88	−0.02	1.01	0.592
Type II EOC	9.19	2.61			

Notes:

* ΔCt miR-141 = (Ct miR-141−Ct miR-16);

** ΔΔCt miR-141 = ΔCt miR-141 Type I EOC-ΔCt miR-141 Type II EOC
